# A Review of Bioinformatics Tools for Bio-Prospecting from Metagenomic Sequence Data

**DOI:** 10.3389/fgene.2017.00023

**Published:** 2017-03-06

**Authors:** Despoina D. Roumpeka, R. John Wallace, Frank Escalettes, Ian Fotheringham, Mick Watson

**Affiliations:** ^1^The Roslin Institute, Royal (Dick) School of Veterinary Studies, The University of Edinburgh,Edinburgh, UK; ^2^The Rowett Institute of Nutrition and Health, Department of Life Sciences and Medicine, University of Aberdeen,Aberdeen, UK; ^3^Ingenza Ltd, Roslin BioCentre,Midlothian, UK

**Keywords:** metagenomics, bioinformatics, next generation sequencing, assembly, gene prediction, bioprospecting

## Abstract

The microbiome can be defined as the community of microorganisms that live in a particular environment. Metagenomics is the practice of sequencing DNA from the genomes of all organisms present in a particular sample, and has become a common method for the study of microbiome population structure and function. Increasingly, researchers are finding novel genes encoded within metagenomes, many of which may be of interest to the biotechnology and pharmaceutical industries. However, such “bioprospecting” requires a suite of sophisticated bioinformatics tools to make sense of the data. This review summarizes the most commonly used bioinformatics tools for the assembly and annotation of metagenomic sequence data with the aim of discovering novel genes.

## Background

The term microbiome refers to the entire community of micro-organisms that exist within any particular ecosystem, and includes bacteria, archaea, viruses, phages, fungi, and protozoa; though the majority of microbiome studies focus only on the bacteria and archaea. There are two main methods for studying the microbiome using high-throughput sequencing: marker-gene studies and whole-genome-shotgun (WGS) metagenomics. In marker-gene studies, generic primers are designed to PCR amplify a particular gene (e.g., 16S rRNA for bacteria/archaea, 18S for fungi) from all genomes present in a sample, and the resulting product is sequenced. The sequences are clustered into operational-taxonomic-units (OTUs) and these are compared across samples. Whilst fast and cheap, this method does not reveal anything else about the hundreds of thousands of genes encoded in the parts of the (meta) genomes that remained unsequenced.

Metagenomics, also referred to as WGS- or shotgun- metagenomics, can offer an alternative and complementary method. Handelsman et al. (1998) first coined the term as the functional analysis of a collection of microbial DNA extracted from soil samples. Metagenomics refer to the application of sequencing techniques to the entirety of the genomic material in the microbiome of a sample. Crucially, by sequencing the genomes of all organisms rather than a single marker gene, metagenomic studies can provide information about the function of genes, the structure and organization of genomes, identification of novel genes and biocatalysts, community structure and evolutionary relationships within the microbial community.

Advances in metagenomics have themselves been driven by advances in second- and third- generation sequencing technologies, which are now capable of producing hundreds of gigabases of DNA sequenced data at a very low cost (Watson, 2014). The high sequencing depth offered by such advances, means that even the least abundant microorganisms in an environment is possible to be represented. Modern sequencing technologies, in combination with continuing improvements in bioinformatics, have made metagenomic analysis an approachable, affordable and fast technique for most labs.

The microbiome can potentially provide a wide range of novel enzymes and biocatalysts with major applications in the marketplace, for example the biotechnology, biofuels and pharmaceutical industry (Cowan et al., 2004). Hess et al. (2011), through an extended metagenomic study, reported over 2.5 million novel genes and identified more than 27,000 putative carbohydrate-active enzymes with cellulolytic function. They also revealed the nearly complete genomes of 15 microorganisms which had never cultured in the lab. Samples were taken from the rumen of fistulated cows and sequenced using Illumina sequencing. The data were assembled using a *de novo* assembler and screened against public databases to define novelty. Wallace et al. (2015) also sequenced ruminal digesta samples using Illumina sequencing, assembling the data *de novo*. Annotation of the resulting contigs revealed over 1.5 million putative genes, with 58% having no known protein domain. Of over 2700 genes associated with methane emissions, only 0.6% had an exact match in the non-redundant protein database of the NCBI (Roehe et al., 2016).

Venter et al. (2004) discovered over 1.2 million unknown genes using metagenomic sequencing of the Sargasso Sea. Genomic libraries were sequenced, assembled into scaffolds and annotated using gene prediction software and sequence similarity tools. These data were estimated to be derived from more than 1800 different species including many newly discovered bacterial groups. Similarly, the global ocean sampling survey (Sunagawa et al., 2015) described 40 million non-redundant sequences from over 35000 species, only 0.44% of which overlapped with known reference genomes, highlighting the huge “unexplored genomic potential in our oceans.”

**FIGURE 1:**
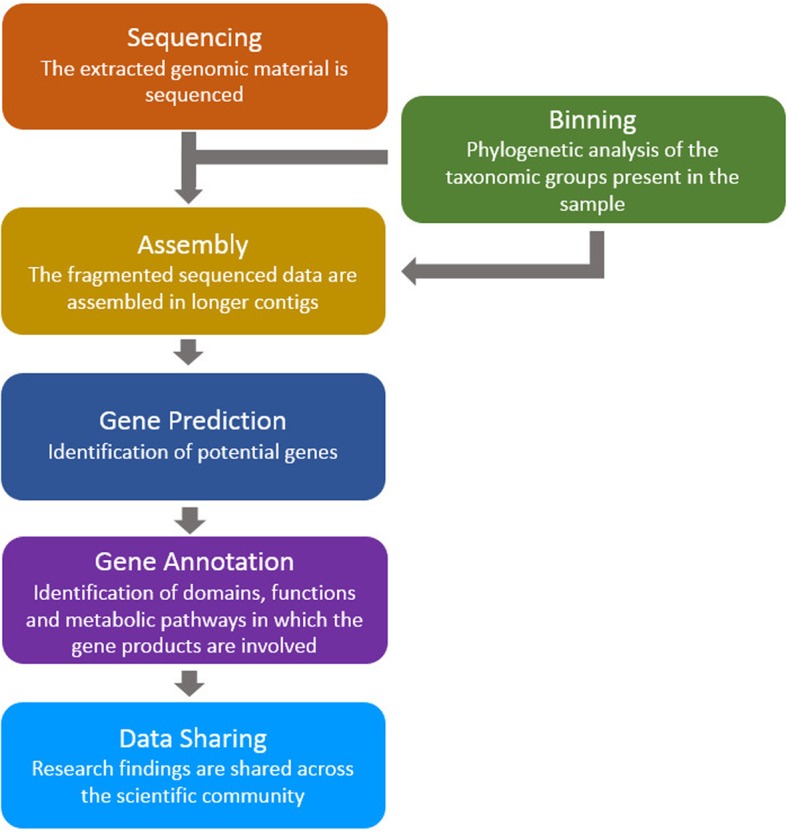
**A typical bioinformatics pipeline.** The genomic material (taken directly from the environmental sample) is sequenced and processed using assembly, gene prediction and gene annotation tools. Finally, the findings are shared between the scientific groups around the world.

The above studies, and many others like them, used similar bioinformatics analysis pipelines: (a) the assembly of sequenced data (directly from environmental samples) in order to construct contiguous sequences (contigs and scaffolds), (b) the prediction of genes (and putative proteins) based on the assembled data, and (c) prediction of domains, functions and pathways for the putative proteins (**Figure 1**). Here, we review a collection of tools for the analysis of metagenomic microbiome sequence data with a focus on the prediction of novel genes and proteins.

## Sequencing Technologies For Whole Genome Shotgun Metagenomics

Many microbiomes are incredibly complex – for example, Hess et al. (2011) estimated that a single cow rumen contains approximately 1000 OTUs – and therefore any sequencing technology applied to microbiome samples needs to be sufficiently deep and comprehensive to capture representative sequences from all species within a microbiome, many of which exist at varying abundances.

Second and third generation sequencing technologies [collectively called “next-generation sequencing,” (NGS)] have enabled much deeper and more comprehensive studies of microbiomes. Second-generation sequencing includes technologies such as Illumina and Ion Torrent that produce many millions of short reads (150–400 bp); whereas third-generation sequencing includes PacBio and ONT which produce much longer reads (6–20 kb) but far fewer reads per run (typically hundreds of thousands).

Illumina technology uses the sequence-by-synthesis method. Short DNA fragments are attached to a glass slide or micro-well and amplified to form clusters. Fluorescently labeled nucleotides are washed across the flowcell and are incorporated complementary to the DNA sequence of the clustered fragment. Fluorescence from the incorporated nucleotides is detected, revealing the DNA sequence. Illumina is almost certainly the leading sequencing technology in genomics labs. It offers the highest throughput, producing relatively short reads with length up to 300 bp, and with the lowest cost per-base. The Illumina output is compatible with the most applications for further study (van Dijk et al., 2014).

In Ion Torrent technology, DNA fragments are attached to beads, and single beads are placed into micro-wells. Each one of the four nucleotides flows through the wells and gets incorporated into a complementary strand, and in doing so, releases an H^+^ ion that can be measured as a voltage change. This process is repeated in multiple cycles. The Ion Torrent technology can finish a run in a significantly less time than other platforms and produces reads up to 400 bp length. However, it is not as widely used as Illumina technologies possibly due to the high rate of homopolymer errors (van Dijk et al., 2014).

Pacific Biosciences is based on SMRT sequencing technology. An engineered DNA polymerase is attached to a single strand of DNA, and these are placed into micro-wells called ZMWs. Each of these ZMWs contains a polymerization complex of a sequencing primer, the template and a DNA polymerase attached to the bottom. During polymerization, the incorporated phospholinked nucleotides carry a fluorescent tag (different for each nucleotide) on their terminal phosphate. The tag is excited and emits light which is captured by a sensitive detector (through a powerful optical system). At the end, the fluorescent label is cleaved off and the polymerization complex is ready for extending the strand (Buermans and Den Dunnen, 2014). The PacBio sequencing platforms require a large amount of genomic DNA as input; however, the platforms are capable of very long reads (10–15 kb with some reads >50,000 bp; Goodwin et al., 2016). PacBio sequencing has a high raw error rate (∼15%) but this can be corrected to very high accuracy (Koren et al., 2012; Chin et al., 2013).

Oxford Nanopore technologies also offer single-molecule sequencing. In nanopore sequencing, a single strand of DNA passes through a protein nanopore and changes in electric current are measured. The DNA polymer complex (used in this technology) consists of a double stranded DNA and an enzyme which unwinds the double strand and passes the single stranded DNA through the nanopore. As the DNA bases pass through the pore, there is a detectable disruption in the electric current and the order of the bases on the DNA stand is identified. In 2014, ONT released the MinION sequencing systems which, unlike the other technologies bulk sequencing installations, is a palm-sized device producing long reads in real time. At launch, the MinION read length was approximately 6–8 kb (Jain et al., 2015; Loman and Watson, 2015); however, Urban et al. (2015) published a lab protocol which could improve the MinION reads length producing many reads even longer than 100 kb. Like PacBio, ONT technologies also have high systematic error rates (Ip et al., 2015).

## Metagenomic Assembly

DNA sequencers sequence fragments of genomes, and assembly refers to the process of reconstructing *in silico* the original genome sequence from the smaller sequenced fragments. Assembly of a single genome is a relatively complex procedure as repetitive elements, within genomes, make the assignment of reads to chromosomes non-trivial [reviewed in Nagarajan and Pop (2013)]. So-called “*de novo*” assemblers use a reference-free strategy for constructing contiguous sequences (contigs). *De novo* assembly software tools use one of two main paradigms: OLC or the *de Bruijn* graph approach. Both algorithms are based on graphs consisted of nodes connected with edges. In the OLC approach, all reads are compared pair-wise to find regions with significant overlaps. The overlapping reads are combined into a graph and the result can be used to reconstruct longer contiguous consensus sequences. OLC assemblers tend to be very accurate; however, comparing each read with every other read is computationally expensive, and doesn’t work well for short reads. Many more recent *de novo* assemblers use the *de Bruijn* graph approach (Pevzner et al., 2001) which constructs a graph by reading the consecutive kmers (sequences of k bases long) within each read. Again, the resulting graph can be used to construct longer, contiguous genome sequences. The advantage of the *de Bruijn* graph is that it can be constructed without pairwise comparison and is, therefore, computationally less expensive than OLC approaches; however, due to the use of kmers, *de Bruijn* graphs are very sensitive to sequence errors, and the (often) relatively short kmers used can result in false joins between sequences.

There are some standard statistical measures for evaluating the performance of assembly tools. These often refer to the number of scaffolds, their length, cover rate (the proportion of the genome covered by assembled scaffolds) and gene prediction/completeness (using gene predictors in later stage). One of the most useful assembly measures is the N50 size, defined as the scaffold length value such that 50% of the assembled sequences are equal or longer (Mäkinen et al., 2012). Contig and scaffold lengths are particularly important metrics for bio-prospecting as these need to be longer than gene-length to enable full length recovery of the gene sequence. MetaQUAST (Mikheenko et al., 2016) is a tool specifically designed for the quality assessment of metagenomics assemblies. Amongst other things, MetaQUAST uses alignment of the original reads to the assembled data to enable detection of putative structural variants and mis-assemblies.

Metagenomic assembly refers to the simultaneous assembly of all genomes within a metagenomic sample, and is clearly more complex than single genome assembly. Due to the data sizes involved, most current metagenomic assemblers use a *de Bruijn* graph data structure for assembly. MetaVelvet (Namiki et al., 2012) is a metagenomic *de novo* assembler, extending the single-genome assembler Velvet (Zerbino and Birney, 2008). There are two main steps in MetaVelvet. First, for given set of metagenomic reads, a large *de Bruijn* graph is constructed; and second, this mixed *de Bruijn* graph is decomposed into subgraphs so that each subgraph represents one “species” or genome/chromosome. The coverage difference between nodes (coverage is defined as the number of reads that contribute to a node) and the connectivity of the nodes are used to distinguish the different subgraphs. MetaVelvet authors reported longer N50 sizes, higher cover rates of genomes (compared to other metagenome and single genome assemblers) and high numbers of predicted proteins (by MetaGene gene finding software, Noguchi et al., 2006). However, the chimera rates (number of wrongly associated points in assembly networks) of MetaVelvet are slightly higher than other assemblers. MetaVelvet performs better than single genome assemblers when using short reads. An extension of MetaVelvet in assembling metagenomics data is MetaVelvet-SL (Sato and Sakakibara, 2015) which focuses on identifying and classifying chimeric nodes in the assembly network. The authors report that the MetaVelvet suite of tools outperform some commonly used assemblers such as IDBA-UD (Peng et al., 2012) and Ray Meta (Boisvert et al., 2012).

In IDBA-UD (Peng et al., 2012), contigs are constructed through progressive cycles of assembly using gradually increasing *k*-mer values. Starting with the minimum *k*-mer value, the first *de Bruijn* graph is constructed for a set of input reads. The output contigs, constructed with a fixed *k*-mer value, *k*_i_, are used as input for the construction of the *de Bruijn* graph with *k*-mer value *k*_i+1_. Therefore, the output of a previous iteration is used as input for the following one. Each cycle incorporates an error correction step, and a progressive depth threshold is used to separate low from high depth contigs. The final scaffolds are constructed based on the outputted contigs in combination with paired-end reads information. Metagenomic assembly with IDBA-UD, in real and simulated data, showed also N50 values, high contig length and large number of predicted genes (by MetaGeneAnnotator, Noguchi et al., 2008). The major innovation of IDBA-UD is the iteration of *k*-values in cycles of increasing *k*-mer size, followed by a local assembly process. The increasing *k*-mer size in cycles contributes to less branches in the assembly network and longer contigs while the local assembly reduces the gaps and resolves repeats in the *de Bruijn* graph. However, iterating over many *k*-mer values requires more computational resources (time and memory). Megahit (Li et al., 2015) uses a very similar approach to IDBA-UD, but takes advantage of succinct *de Bruijn* graphs (Bowe et al., 2012) and GPUs, which lowers the memory requirements and increases speed, respectively.

Ray Meta is a scalable software tool that uses distributed computing and the MPI to handle large datasets. The assemblies are constructed based on *de Bruijn* graphs. The average coverage depth is calculated through parallel assembling processes by local coverage distributions of the *k*-mers (Boisvert et al., 2012). The assembled data are validated by aligning them against reference genomes [MUMmer software (Kurtz et al., 2004)]. Ray Meta can distribute the assembling process of large metagenomic data into multiple cores minimizing run time and memory requirements (Boisvert et al., 2012). By using high-performance computing (HPC), Ray Meta is able to handle large amounts of data; assembling them in less time and performing better than MetaVelvet (Namiki et al., 2012) in assembling simulated bacterial data (from human samples).

Based on *de Bruijn* graphs assembly, Pell et al. (2012) described a probabilistic method for storing *de Bruijn* assembling graphs using less memory. Bloom filters are probabilistic data structures which test the membership of an element in a dataset, allowing false positives but no false negatives (Bloom, 1970). This method uses bloom filters for storing large *de Bruijn* assembly graphs. A range of false positive rates is available for controlling memory requirements. Additionally, the authors used a memory efficient partitioning method which allows division of the *de Bruijn* graph into disconnected sub-graphs that can be assembled separately. Each of the sub-graphs represents a separate clade within the metagenomic sample. Allowing a higher false positive rate decreases the graph storage requirements and for higher false positive rates the partitioning strategy can handle more elaborate local assemblies. A fixed memory data structure allows prediction of the expected false positive rate as more data are added. Different available *k*-mer sizes can be used; however, the memory usage is independent to the *k*-mer size chosen. Finally, memory efficient partitioning can create separate sub-sets based on common features of the data.

MetAMOS (Treangen et al., 2013) is an example of a modular framework which combines existing tools into a metagenomic analysis pipeline. The pipeline is divided into three steps: In the first step, (meta) genome assembly is performed with a choice based on the sequencing technology used. Secondly, scaffolds are created using paired-end and mate-pair data using Bambus 2 (Koren et al., 2011). Finally there is a post-assembling stage where the scaffolds are annotated and taxonomically identified. Choosing the appropriate assembler for a specific application can be difficult, and (as mentioned above) assembly tools vary in performance. A major advantage of MetAMOS is the ability to test multiple assembly tools and give the opportunity to choose the most appropriate for a given dataset. Interestingly, using a combination of assemblers within MetAMOS appears to improve performance (contig length, contiguity, and error rates). One of the key features for maintaining the contiguity of the scaffolds is the identification of genetic variation patterns. MetAMOS is able to maintain a contiguous genomic backbone whilst also highlighting variable regions. An HTML report is produced summarizing the results of the analysis at the end.

## Phylogenetic Binning

Binning is the process of clustering genomic sequences into groups so that each subset represents a separate biological taxon. Binning and assembly are two related procedures – binning can be performed pre-assembly, or integrated into the assembly process; in either scenario, binning attempts to prevent co-assembly of mixed genomes. In theory, each bin represents a single genome and is assembled separately, removing some of the problem of incorrect assemblies connecting contigs from diverse taxa.

LikelyBin is an un-supervised statistical approach for binning metagenomic fragments. The method uses a Markov Chain Monte Carlo approach and is built on the assumption that the oligonucleotide frequency distribution is homogeneous within a bacterial genome. This is an over-simplification and regions that break the assumption (such as horizontal gene transfer islands) need more complicated statistical models. LikelyBin uses an “index of separability” between genomes based on the *k*-mer distributions. The method is reported to perform well in low complexity metagenomic communities (Kislyuk et al., 2009).

PHYSCIMM (Kelley and Salzberg, 2010) combines Phymm (Brady and Salzberg, 2009) and SCIMM (Kelley and Salzberg, 2010). Phymm uses IMMs trained on known genomes to classify the data; whereas SCIMM is a totally un-supervised tool, also based on IMMs. The first stage of PHYSCIMM is to partition the classified sequences by Phymm and then use SCIMM on the unclassified data. The authors reported that the contribution of the supervised step is important in binning complex samples (containing many microbial species) since the un-supervised clustering stage is improved when the supervised stage precedes. PHYSCIMM can thoroughly describe the microbial composition of a sample when the species are represented in public databases. Choosing the classification level is required for clustering while there are guidelines to help the user set the software parameters.

In MetaWatt (Strous et al., 2012), four steps are carried out. The first step is metagenomic assembly and Strous et al. (2012) use MetaVelvet. Secondly, the assembled contigs are clustered/binned according to observed tetranucleotide frequencies. Thirdly, the bins created in step two are inspected for taxonomic signatures (using BLAST) and for sequence coverage. Good bins were those that had a consistent taxonomic profile and similar within-bin coverage. Finally, these bins are used to build IMMs and each contig is assigned to the bin with the highest score. MetaWatt is an open source algorithm that can be implemented in any platform which supports BLAST and Glimmer. It is potentially scalable (due to less running time) and able to handle large amounts of sequence data. MetaWatt may be attractive to researchers who are not bioinfomaticians as it is available through a graphical-user-interface (GUI). This allows the user to view and choose the bins for IMM modeling. The graphics can be exported in SVG format and the bins as FASTA for further analysis and annotation.

CONCOCT (Alneberg et al., 2014) is a binning program which uses GMMs, sequence composition and the coverage across multiple samples for clustering metagenomic data. A Bayesian approach (automatic relevance determination, Corduneanu and Bishop, 2001) is used for determining the number of clusters. After assembling the sequenced reads, the longer contigs are fragmented and the reads are mapped back onto contigs to determine coverage across all samples. The coverage and sequence composition vectors are joined to form a combined profile for each contig, and a GMM can be used to describe the entire dataset. CONCOCT was tested using mock and real metagenomic data. The precision of CONCOT on the mock data was very high while the majority of clusters were highly consistent (mostly consisting of contigs from the same species). CONCOCT was reported by the authors to perform well in clustering complicated microbial communities. However, some strain specific variations were difficult to resolve. This limitation is probably due to the low coverage of some contigs in the sample making the formation of distinct clusters difficult. A very interesting application of CONCOCT was reported in Alneberg et al. (2014) for the reconstruction of pathogenic genomes from real fecal data taken from the Shiga toxin-producing *E. coli* outbreak in 2011. The software seems to identify and cluster pathogenic microbial genomic material together. Additionally, it managed to distinguish protective microbial genomes and present them as distant to the pathogenic ones. Thus, CONCOCT has been suggested for extracting biologically important information and could possibly contribute to recovery after infection.

Latent strain analysis is a pre-assembly algorithm which aims to bin short sequenced reads into microbial categories. This method is based on the assumption that reads which belong to the same organism are expected to have the same coverage across samples. LSA uses *k*-mer frequencies and clustering to cluster sequences, and can be applied to very large datasets in fixed memory. The LSA output can be used for *de novo* assembly or taxonomic mapping and it is capable of handling datasets as large as hundreds of Gb (Cleary et al., 2015).

## Metagenome Gene Prediction

Annotating the assembled data and identifying genomic features such as genes and regulatory elements is the next step in a metagenomic analysis pipeline. Usually, the short reads produced by NGS are difficult to be assembled and even after assembly, contigs and scaffolds can often be short and fragmented. MetaGeneAnnotator (Noguchi et al., 2008) is a metagenomic gene-finding algorithm which predicts genes on short sequences from un-characterized metagenomic communities based on the assumption that CG content correlates with di-codon frequencies. The software can automatically detect prophage genes through implemented statistical models as well as chromosomal backbone prokaryotic genes. It can also predict translation starting points by using RBS models. An interesting feature of MetaGeneAnnotator is the RBS map output which, apart from the gene location, gives information for translation initiation mechanisms useful for the analysis of evolutionary relationships (Noguchi et al., 2008).

Orphelia is available as both a web-server and command-line tool, and uses a two-step machine learning approach. In the first step, linear discriminant analysis based on monocodon usage, dicodon usage and translation initiation sites is used to extract features from genomic sequence. In the second step, an artificial neural network is constructed, combining the features from step 1 with information on open reading frame length and GC-content to compute the probability that an ORF (Open-reading-frame) encodes a protein. Orphelia was shown to demonstrate higher specificity but lower sensitivity in gene prediction compared to MetaGeneAnnotator and MetaGene (Noguchi et al., 2006, a precursor to MetaGeneAnnotator) on simulated data.

Glimmer-MG (Kelley et al., 2012) is an extension of the popular bacterial gene-prediction software Glimmer (Delcher et al., 2007). Glimmer-MG starts by clustering data which likely belong to the same organism, using Phymm (Brady and Salzberg, 2009); uncategorized data are then clustered using Scimm (Kelley and Salzberg, 2010). Gene models, based on HMMs, are trained within each cluster, incorporating probabilistic models for gene length and start/stop codons, and used to predict genes. The authors report that the combination of gene prediction with phylogenetic classification results in more accurate predictions. In simulated data, Glimmer-MG identifies insertions/deletions more accurately than FragGeneScan; and can also predict substitution errors affecting stop codons. In both real and simulated data, Glimmer-MG predicted genes in error-prone sequences more accurately than other methods.

FragGeneScan (Rho et al., 2010) is designed to predict genes (often fragmented) directly from short reads themselves, without the need of assembly; however, the software can also run on assembled sequenced. FragGeneScan uses hidden Markov models (HMMs) trained with sequencing error and codon usage models. Sequencing errors may produce frameshifts which, in many cases, result in fragmented genes that are difficult to identify. The major feature of FragGeneScan is the inclusion of sequencing error models into six-periodic inhomogenous Morkov models. FragGeneScan presents higher performance in predicting genes than MetaGene and contains a set of parameters for analyzing reads produced by the main NGS technologies. Finally, the authors report that FragGeneScan is less affected by the read length since it achieves consistently high gene prediction performance in a range of read lengths compared to MetaGene.

Finally, Prokka (Seemann, 2014) is a pipeline for annotating bacterial genomes and has an option for highly fragmented metagenomic assemblies. Prokka uses published open-source software tools to predict protein coding and tRNA/rRNA genes. Putative genes and products are annotated by comparison to public databases. Testing Prokka against RAST (Aziz et al., 2008) and xBase2 (Chaudhuri et al., 2008) in annotating E. coli data, Prokka showed overall the best performance. Prokka is freely available, is fast, can be installed on a typical desktop computer and integrated into metagenomic pipelines.

Most of the metagenomic gene annotation tools focus only on bacterial and archaeal genomes; presumably, as this is an easier problem to solve. However, most environmental samples will also contain Eukaryotes, which require different tools and methods due to the presence of introns and the more complex nature of Eukaryotic genomes. GeneMark is an abinitio gene prediction software suite that has modes for both metagenomes (MetaGeneMark, Zhu et al., 2010) and Eukaryotes (GeneMark-ES, Ter-hovhannisyan et al., 2008), though we are unaware of studies that have combined these two. GenScan (Burge and Karlin, 1997) is another popular method used for single eukaryotic genomes, and contains models for exons, introns and intergenic sequences. Whilst gene prediction in single eukaryotic genomes is a very active area of research, we are not aware of any studies demonstrating these on metagenomes, and this may be a fruitful area for future research priorities.

## Protein Domain Databases

There are a large number of published protein sequence/feature/structure databases, each with a different focus and strengths and weaknesses. Many overlap and contain shared information. InterPro (Mitchell et al., 2015) is a collaboration between 12 such databases, and is a single portal for access to information about proteins. Interpro integrates information about domains and active sites, proteins families, and protein activity and function. Each module has its own strengths and Intepro aims to combine all these resources for better characterization of query sequences. Protein families, domains and sites are combined in one database, names are checked for consistency and links to original publications are included. Accessible via the web, users may query the database by sequence or name, and InterPro searches for possible matches. If the query sequence is available in multiple databases, the results are presented in a new window. If there are no matches, then the sequence is passed into InterProScan (Hunter et al., 2012).

InterProScan is a protein function prediction software pipeline that simultaneously searches the 12 member databases of InterPro when given an input query sequence (Hunter et al., 2012). InterProScan is parallelized and can handle millions of sequences. InterProScan uses models of proteins and domains from the InterPro database, and the Phobius analysis algorithm (Krogh et al., 2004) is available as an additional feature. Outputs in several formats are possible (text and images). InterProScan is a very powerful way of predicting protein function/domains/families/active sites, and therefore is an essential tool for bio-prospecting.

## Pathway Databases

The term “pathway” is loosely defined and generally refers to a series of actions between biomolecules that results in a particular product. Reactome (Fabregat et al., 2016) is a free, open-source and curated database of biological pathways. The reactions are organized hierarchically, with single reactions in the lowest level, while interconnected pathways are organized in higher levels (Haw and Stein, 2012). Data stored in Reactome has been extracted from the experimental literature, with information curated by researchers, curators, editors and reviewers. In Reactome also references other databases such as UniProt, Ensembl, KEGG and many others (Consortium, 2012; Flicek et al., 2012; Kanehisa et al., 2012; Brown et al., 2015).

Kyoto Encyclopedia of Genes and Genomes (KEGG) is a database connecting genomic, biochemical and phenotypical information from multiple individual databases (Kanehisa et al., 2008). It contains information about metabolic pathways and the genomes, genes, proteins and enzymes that contribute to those pathways; as well as details about genetic and environmental processes, diseases and drugs pathways. There are many links to external databases such as NCBI Entrez Gene, OMIM and UniProt^1,2^. Unfortunately, in 2011, FTP access to KEGG was ceased, and KEGG is now only accessible through the website and via a series of API (application program interfaces). This limits the ability of tool builders to integrate KEGG into their pipelines.

WikiPathways is an open source project different to the other pathway databases (Kelder et al., 2012). It is part of the MediaWiki software and relies on creation, curation and editing of various biochemical pathways by any user with a WikiPathways account (Pico et al., 2008). WikiPathways contains many different signaling pathways involved in different biological processes across many species. WikiPathways is a new paradigm for storing and organizing large amounts of biological data, relying on community commitment to maintain and curate the data, contributing to the overall success.

Finally, MetaCyc (Caspi et al., 2016) is a large, comprehensive database of pathways and enzymes from across all domains of life, with data coming predominantly from experiments published in the literature. MetaCyc claims to be the largest collection of curated metabolic pathways. In reality, no pathway database is complete, and in some environmental samples fewer than 10% of predicted genes or proteins will map to a known pathway or reaction (Wallace et al., 2015). It is therefore common to use multiple databases and interpret the results collectively.

## Targeted Gene Discovery

Where researchers are only interested in a small number of proteins, it is not always necessary to annotate the entire metagenome. Xander (Wang et al., 2015) is a metagenomic gene-targeted assembler which uses HMMs to guide graph traversal. Xander uses two data structures – a *de Bruijn* graph and a profile HMM – which are used to create a novel combined weighted assembly graph. From any given vertex, Xander can traverse the graph in both directions, finding the best path that corresponds to the provided HMM. This gene-targeted assembly is less compute intensive due to the smaller amount of graph to be explored.

## Data Sharing and Online Portals

Metagenomic assembly, gene prediction and annotation creates large files, often in formats that scientists struggle to open, query and search on standard desktop or laptop computers. Meta4 (Richardson et al., 2013) is a simple web application that allows users to query, search and browse the millions of gene and protein predictions that often result from metagenomic assembly and annotation. An underlying database can be built from common formats such as FASTA and GFF. Meta4 can be installed on any server running Linux, Apache and MySQL and provides a very simple and user-friendly interface. Meta4 includes web-services to access tools such as BLAST and InterProScan. One of the advantages of Meta4 is that it can be set up on a private or institutional server prior to data release and publication.

However, if researchers are happy to make their data public, a number of online all-in-one metagenome annotation portals exist.

MG-RAST (Glass et al., 2010) is a web-based platform providing access to a variety of tools for metagenomic analysis. After removing repetitive sequences and low-quality regions, MG-RAST maps sequence data to three non-redundant databases and creates a phylogenetic profile of the metagenomic sample. Parameters such as similarity and percentage identity, e-value and alignment length can be adjusted. Metabolic and functional profiles are also predicted using novel non-redundant protein databases and public data such as KEGG. Many sequencing technologies are supported and the results are available for sharing and downloading.

EBI MetaGenomics (Hunter et al., 2014) is a system dedicated to metagenomic analysis based at the EBI. The pipeline starts with quality control of the dataset, where the data are trimmed and duplicates are removed. ORFs are predicted using FragGeneScan and then fed in to InterProScan to assign putative function, protein domains, and pathways. Finally, the sequences are taxonomically classified into phylogenetic taxa giving an indication of possible microbial members in the community.

IMG/M (Markowitz et al., 2014) is a comparative metagenomic analysis system built on the IMG platform for microbial genome annotation (Markowitz et al., 2014). IMG/M accepts sequenced data from many sequencing platforms and process them using multiple methods. IMG/M contains datasets from various metagenomic samples as well as all genomes from IMG. Thus, metagenomic samples can be compared based on the abundance of proteins, domains, enzymes, pathways or functional class, and can be integrated with public data. A binning step aims to categorize metagenomic data into phylogenies while characteristics such as phenotype, habitat, living conditions and diseases can also be attributed to the data (Markowitz et al., 2008).

The EDGE platform (Li et al., 2017) also contains a number of relevant software tools including QC, assembly, annotation, taxonomic classification and phylogenetic analysis. These are available through an online portal and modules can be built into custom pipelines. A summary of the tools, databases and technologies described above, alongside relevant features, is provided as **Supplementary Table S1**.

## Conclusion

The microbiome is the community of microorganisms that lives in a particular ecosystem and metagenomics is the process of simultaneously sequencing the genomes of all organisms in a particular biological sample. Advances in sequencing technology have allowed us to assay microbiomes at unprecedented depth using metagenomics. Research into diverse microbiomes has revealed a huge amount of novelty, including genes that encode proteins which may be of significant industrial value. Here, we presented a review of bioinformatics tools that enables researchers to analyze large metagenomic datasets and extract putative novel genes/proteins/enzymes. These may be fed into experimental pipelines for the characterization of protein function and activity, and may provide novel enzymes of significant value.

## Author Contributions

DR, RW, FE, IF, and MW co-authored and proof-read the manuscript. All authors approved the final manuscript.

## Conflict of Interest Statement

The authors declare that the research was conducted in the absence of any commercial or financial relationships that could be construed as a potential conflict of interest.

## Footnotes

1 NCBI Entrez Gene. www.ncbi.nlm.nih.gov/entrez/query.fcgi?db=gene

2 NCBI OMIM. http://www.ncbi.nlm.nih.gov/omim

## Supplementary Material

TABLE S1 **A summary of the tools, databases and technologies used for metagenomic bioprospecting**.

## Abbreviations

GMM: Gaussian mixture model
GPU: graphical processing unit
GUI: graphical user interphase
IMM: Interpolated Markov model
LSA: latent strain analysis
MPI: message passing interface
NGS: next generation sequencing
OLC: overlap layout consensus
ONT: Oxford nanopore technologies
OTU: operational taxonomic unit
PacBio: Pacific Biosciences
RBS: ribosomal binding site
SMRT: single molecule real time
WGS: whole genome shotgun
ZMW: zero-mode waveguide
